# The Effect of Education on Quality of Life in Patients under Interferon Therapy

**Published:** 2010-09-01

**Authors:** Ahmad Ali Asadi Noghabi, Mitra Zandi, Abbas Mehran, Seyed Moayed Alavian, Ali Hasanpour Dehkordi

**Affiliations:** 1 School of Midwifery and Nursing, Tehran University of Medical Sciences, Tehran, Iran; 2 School of Medical Sciences, Tarbiat Modares University, Tehran, Iran; 3 Baqiyatallah Research Center for Gastroenterology and Liver Disease, Baqiyatallah University of Medical Sciences, Tehran, Iran; 4 Faculty of Medicine, Shahrekord University of Medical Sciences, Shahrekord, Iran

**Keywords:** Education, Viral Hepatitis, Quality of Life, Interferon Therapy

## Abstract

**Background and Aims:**

The main purpose of treating and caring for patients with chronic viral hepatitis is to promote life satisfaction and a feeling of well-being in patients suffering from this disease. The aim of this study was to evaluate the effect of education on quality of life in patients with chronic hepatitis who were treated with Interferon alpha.

**Methods:**

This quasi-experimental study was conducted on 60 patients with viral hepatitis. The intervention included teaching them the method of self injection of Interferon alpha 2 b, giving them educational pamphlets and then following their continuing treatment with interferon. Patients were randomly assigned to two 30-patient groups. The data- gathering tool was a demographic characteristics questionnaire and the Quality of Life Questionnaire for Patients with Chronic Liver Disease (CLDQ). The educational program was done in four 45- minute sessions for the case group and their relatives. The follow-up period was 12 weeks. Quality of life in patients with chronic hepatitis was measured before initiating interferon therapy, and after the educational period. Quality of life in the two groups was compared.

**Results:**

The total quality of life score in the two groups before therapy did not show any significant difference (P = 0.351); while 12 weeks after education there was a significant difference between the two groups (P < 0.001) in three items including abdominal symptoms (P = 0.01), worry (P < 0.001) and emotional factors (P < 0.001). The other three items did not show a significant difference between the two groups. The total quality of life score in the case group was significantly different before and after education (P < 0.001), and improved after education. The total quality of life score in the control group did not differ significantly after 12 weeks (P = 0.143).

**Conclusions:**

Planning short and simple educational programs has a significant effect on the patient’s control of his/her disease and its side effects; and can improve quality of life, life satisfaction, and mechanisms of coping with treatment in patients with viral hepatitis.

## Introduction

The World Health Organization (WHO) has reported chronic liver disease to be responsible for 1.4 million deaths worldwide in 2002. Hepatitis C virus (HCV) stands as one of the most important etiologies of chronic liver disease and is an emerging infection in the world and in Iran [1]. It seems that less than 1% of Iranians are infected with HCV infection [2]. The control of HCV is possible and it needs to be eradicated with the best approved drugs [3]. Hepatitis B virus (HBV) infection is also an important health problem worldwide and about 1,400,000-2,000,000 patients suffer from it in Iran [4]. The main effective drugs in the treatment of HCV infection is alpha interferon plus ribavirin and  alpha interferon is also one of the approved therapies in chronic hepatitis B [5][6]. Several studies have documented that impaired health-related quality of life (HRQoL) is associated with chronic hepatitis B and C [7][8]. Studies have shown that the progression of liver disease and ineffective antiviral therapies increasingly affect the quality of life, and the mental and physical well-being of patients [9]. The side effects  of interferon therapy, including an influenza-like syndrome, affect the quality of life of patients as well [10]. The quality of life effects of treatment regimens also affect adherence to treatment regimens [11]. During therapy with alpha-2b interferon, patients should be closely monitored for side effects [10], so appropriate and timely supervision during treatment is necessary to limit these effects [11]. Frequently treatment causes fatigue, muscular pain, influenza-like  symptoms, changes in the mental status and sexual desires of the patient, which will negatively affect the patient’s life, social communication and  abilities [12]. The importance of these side effects forms the basis of the physician’s and the patient’s decision-making regarding the continuation of the therapy, or of the cessation of the course of therapy  before its completion. Termination of therapy before its completion shows the patient’s intolerance [12]. Side effects of Interferon alpha begin a few hours  after its administration and the patient develops tolerance in a few weeks with continuation of therapy [13]. Patient education and effective treatment are the cornerstones for enabling the patient to adhere to a therapeutic regimen. Monitoring the patient and dose adjustment during the treatment period is necessary [14]. At times lack of awareness about the  route of application results in early termination of the treatment [12]; as a result, a study of the education of the patient in the proper method of the self- injection of interferon and the method of familiarizing him or her with the side effects and strategies of coping is essential and will lead to an improved quality of life. This study had been conducted for these reasons.

Our primary goal was to study the effect of education on the quality of life of the patient suffering from chronic hepatitis B and C under treatment with Interferon alpha.

## Materials and Methods

This is a quasi-experimental study in a pretest-posttest method conducted at Tehran Hepatitis Center- Baqiyatallah Research Center for Gastroenterology and Liver Diseases. Sampling was nonrandomized and based on sample characteristics (age 18-60 yrs, absence of other infections and chronic diseases, first time treatment for Interferon alpha therapy and absence of cirrhosis). Sixty patients were randomly assigned to the two 30-patient groups of cases and controls. Following referral to Tehran Hepatitis Center, patients fulfilling our selection criteria and willing to participate in the study were assigned to the case and control groups one after the other. The Quality of Life Questionnaire for Patients with Chronic Liver Disease (CLDQ) and the demographic information of the patients were filled out anonymously and codes were used with the help of the researcher.

The CLDQ is the first tool to assess quality of life in chronic liver disease patients reasonably adjusted to include fatigue, activity, emotional symptoms, abdominal symptoms, systemic symptoms and worry. The scores are graded from 1 to 7 for each category making the minimum possible scores 29 and the  maximum 203. The validity and reproducibility of this questionnaire had been previously studied in Iran [15][16]. The eliability of the CLDQ was measured by internal consistency using Cronbach’s alpha test with a reported consistency of over 95%.

Cases were asked to write down their free time on the back of the questionnaire to set up times for their education classes. These classes were held  once a week and in each class educational pamphlets were distributed among the cases. Follow-up of the  patients was done as self-reporting with in-person attendance every month. Each educational session had a maximum of 6 patients and 6 accompanying persons. These accompanying persons had a supportive care role. The sequence of the classes was as follows:

In the first session, the goals of our study, the nature  of their disease, transmission routes, the diagnosis and treatment of their disease were explained and pamphlets were distributed to the patients. In the second session, the effect of interferon therapy on their disease, the frequent side effects after injection, methods of protecting themselves and controlling these side effects were explained and pamphlets were distributed. In the third session, the method of the self-injection of interferon was explained and the  patients’ questions were answered. A pamphlet was also given out. In the fourth session, the injection of interferon by the patient was observed and their problems, if any, were corrected. Educational sessions were held for a month and patients were followed for 12 weeks. At the time of entering the study and 12 months after initiation of therapy, patients were asked to fill the quality of life questionnaire in both  groups (case and control). The results were analyzed by SPSS (Version 13) using chi-square, Fisher’s exact,Mann-Whitney and Wilcoxon tests. For ethical issues, after the study, the educational pamphlets were also distributed to the control group and the correct method of injection of interferon was also taught to them.

## Results

The demographics data of the patients is shown  and compared in Table 1. As is shown, no significant difference was observed between the two groups in this regard.

**Table 1:** Demographic information of our studied population

	**Control Group**	**Case Group**	**P-value**	**Statistical Test**
Age	37.2 ± 9.5	40.3 ± 14.9	0.33	t- test
**Sex (Percent)**			0.381	chi-square
Male	24 (80%)	22 (73.3%)		
Female	6 (20%)	8 (26.7%)		
**Level of Education**				fisher's exact
Illiterate	0	3 (10%)	0.19	
Primary and Guidance School	15 (50%)	16 (53.3%)		
Diploma and Higher	15 (15%)	11 (36.7%)		
**Marital Status**				chi-square
Single	15 (50%)	13 (43.3%)	0.398	
Married		15 (50%)	17 (56.6%)	
**Number of Children**			chi-square	
3 or less	28 (93.3%)	23 (76.7%)	0.145	
More tdan 3	2 (6.7%)	7 (23.3%)		
**Occupation**			fisher's exact	
Worker	7 (23.3%)	4 (13.3%)	0.076	
Employee	4 (13.3%)	6 (20%)		
Housekeeper	4 (13.3%)	4 (13.3%)		
Student	0	6 (20%)		
Retired	15 (50%)	10 (33.3%)		
**Duration of Disease**				chi-square
1-3 yr	22 (73.3%)	21 (70%)	1	
3-6 yr	4 (13.3%)	5 (16.7%)		
More tdan 6 yr	4 (13.3%)	4 (13.3%)		
**Hospitalization**				fisher's exact
None	28 (93.3%)	30 (100%)	0.492	
Once	2 (6.72%)	0		
**Hepatitis Type**				chi-square
Hepatitis C	20 (66.7%)	24 (80%)	0.243	
Hepatitis B	10 (33.3%)	6 (20%)		

The Quality of Life Questionnaire for Chronic Liver Disease included 6 items: fatigue, activity, emotional symptoms, abdominal symptoms, systemic symptoms and worry, which were separately analyzed  in the two groups before and after our intervention.Quality of life scores before and after intervention  within groups has been shown in Table 2. Quality of life scores before and after intervention between the two groups has been shown in Table 3.

**Table 2:** Quality of Life scores before and after 12 weeks within groups

		**Control **				**Wilcoxon Test**	**Case**				**Wilcoxon Test**
		**Before**		**After**			**Before**		**After**		
**Scores**	Min-Max	Mean	SD	Mean	SD	P	Mean	SD	Mean	SD	P
**Abdominal Symptoms**	3-21	15.9	5.3	15.9	5.6	0.48	17.7	3.1	19.5	3.2	0.00
**Activity**	3-21	19.8	1.9	18.7	2.7	0.01	20	1.9	18	3.6	<0.001
**Fatigue**	5-35	23.4	8	23	7.2	0.68	26.3	6.3	26	6.9	0.08
**Systemic Symptoms**	5-35	28.5	5.2	26.4	6.6	0.03	29.9	4.1	29.1	5.1	0.29
**Emotional**	8-56	33.3	9.9	33	9.2	0.03	40.1	9.2	46.5	10.6	<0.001
**Worry**	5-35	22.3	6.8	21.9	7.4	0.21	24.1	5.3	30.2	6.3	<0.001
**Total Score**	29-203	154.5	28.5	136.9	30.6		158.6	21.4	170	23.6	

**Table 3:** Quality of Life scores before and after 12 weeks between two groups

		**Before Intervention**				**Mann-Whitney Test**	**After Intervention**				**Mann-Whitney Test**
		**Control**		**Case**			**Control**		**Case**		
**Scores**	Min-Max	Mean	SD	Mean	SD	P	Mean	SD	Mean	SD	P
**Abdominal Symptoms**	3-21	15.9	5.3	17.7	3.1	0.43	15.9	5.6	19.5	3.2	0.94
**Activity**	3-21	19.8	1.9	20	1.9	0.8	18.7	2.7	18	3.6	0.08
**Fatigue**	5-35	23.4	8	26.3	6.3	0.26	23	7.2	26	6.9	0.84
**Systemic Symptoms**	5-35	28.5	5.2	29.9	4.1	0.35	26.4	6.6	29.1	5.1	0.04
**Emotional**	8-56	33.3	9.9	40.1	9.2	0.006	33	9.2	46.5	10.6	0.8
**Worry**	5-35	22.3	6.8	24.1	5.3	0.06	21.9	7.4	30.2	6.3	0.64
**Total Score**	29-203	154.5	28.5	158.6	21.4		136.9	30.6	170	23.6	

The systemic symptoms of the control group  were significantly reduced after 12 weeks (P = 0.04) but other aspects including abdominal symptoms (P = 0.94), fatigue (P = 0.84), activity (P = 0.08), worry (P = 0.64) and emotional symptoms (P = 0.8) did not show a significant change after 12 weeks, in the control group.

In the case group there was a significant decrease in abdominal symptoms (P < 0.001), activity (P < 0.001), worry (P < 0.001) and emotional symptoms (P < 0.001) after our intervention but no significant  change was observed in systemic symptoms and fatigue in this group.

Using the Mann-Whitney test, we found a difference in three aspects between the cases and the controls after our intervention including abdominal symptoms (P = 0.01), worry (P < 0.001) and emotional symptoms(P < 0.001). The other aspects did not show any significant difference between the  two groups.

The minimum quality of life score was 29 in this study and the maximum was 203. The mean quality of life scores in our controls was 154.5 before the study and 136.9 after 12 weeks but the Wilcoxon test did not show significant change between the two (P = 0.143).

In our cases, a quality of life score of 158.6 before intervention had increased to 170 after 12 weeks, which was found to be significant using the Wilcoxon  test (P < 0.001). Our data show that before treatment there had not been a significant difference between the two groups in quality of life scores (P = 0.351) but after intervention this difference becomes significant (P < 0.001).

## Discussion

In this study the mean quality of life score in our  controls was decreased after 3 months from 154 to 137 which had been shown in other studies. In 2005, a study of different antiviral treatments on patients with hepatitis C by Kang and his colleagues showed that all treatment regimens had reduced the quality of life of patients in the primary stages of treatment and side effects had reduced their compliance and adherence to treatment; whereas providing information to the patients about their treatment and its side effects will increase their tolerance, compliance and adherence to antiviral therapy [17]. In our cases, the mean quality of life score increased to 170 from its original 158 after three months. This increase is in accordance with previous studies on patients with liver disease in Tehran and Shiraz [16][18].

Certain aspects in our control group did not  show an increase after 12 months while the systemic symptoms showed a decrease during follow up.  Previous studies show that the quality of life of the patient under antiviral treatment decreases in 12-24 weeks after initiation of therapy and will eventually return to its baseline measurement after 24 weeks [12]. Fatigue is a frequent side effect of hepatitis  treatment and may result in early termination of antiviral therapy [12].

In our cases, there was an increase in the scores of abdominal symptoms, worry and emotional symptoms after 12 weeks. In our study, the quality of life score in our cases showed a significant increase after 12 weeks compared to the controls.

It seems that using simple measures against side effects like adequate hydration, light-to- moderate physical activity, scheduling the treatments for times when patients have less work or on weekends, using sedatives, antipyretics and similar measures will  greatly help the control of side effects which will consequently increase the satisfaction and quality of life of patients and ultimately result in tolerance of the treatment [19].

One of the drawbacks of this study is the limited number of the population studied, which should be kept in mind in drawing a final conclusion.  Another point to be considered is the different treatment regimens in hepatitis B and C (Interferon alpha and Ribavirin in hepatitis C and Interferon alpha in hepatitis B) which was a factor not taken into account in this study. The effect of different treatment regimens on hepatitis B and C could be the subject of a separate study. Emotional symptoms were different before intervention in the case and control groups, but a significant increase in the quality of life score could justify a faster and greater increase in the emotional aspect.

## Conclusions

This study showed that continuous education and follow up in chronic hepatitis B and C patients under Interferon alpha treatment could greatly increase their adherence to interferon treatment and decrease the side effects, ultimately resulting in a better quality of life.
